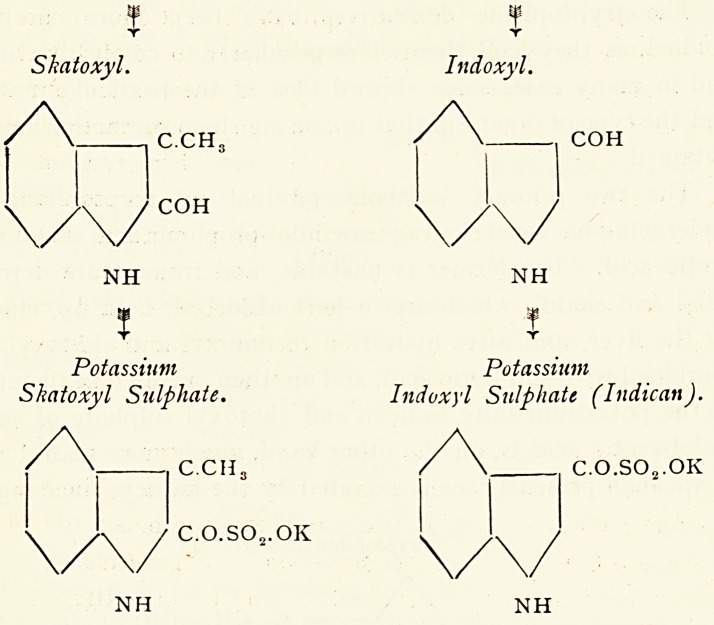# Urinary Products of Intestinal Intoxication
^1^A paper read before the Bath and Bristol Branch of the British Medical Association on April 30th, 1913.


**Published:** 1913-06

**Authors:** G. Hely-Hutchinson Almond

**Affiliations:** Hon. Pathologist to the Royal Mineral Water Hospital, Bath.


### URINARY PRODUCTS OF INTESTINAL INTOXICATION.[1]

BY

G. Hely-Hutchinson Almond, M.A., B.M., B.Ch. Oxon.
*Hon. Pathologist to the Royal Mineral Water Hospital, Bath.*

I propose to discuss the methods, the uses and the limitations of urinary analysis as an index of the excessive or perverted bacterial activity of the intestinal tract.

During the process of normal digestion the food is split up

[1] A paper read before the Bath and Bristol Branch of the British Medical Association on April 30th, 1913.

into simpler molecules, which can be readily absorbed by the intestinal mucosa, and which are there, in the liver, or in some other organ, prepared for absorption into the complex activity of protoplasm. Many of these products are in themselves poisons, and have to be re-combined or re-synthesised into harmless products. For this protective action the liver is mainly responsible, and as we shall presently see, it also takes a large part in detoxicating the products of bacterial energy.

The necessity of bacterial life in the intestine has been a matter of much controversy; but we do know that polar animals can exist and thrive with an alimentary canal that is practically sterile. Where germs freely exist, however, as thev do in all other climates, the presence of bacteria in the intestine seems on the whole to be an advantage, for (under ordinary conditions) certain groups of organisms become immune to us and we to them, and they do their best to keep out the wild intruder by making his existence difficult. Under certain conditions, however, the flora may become abnormal, and when this occurs various forms of intoxication may result.

For the subject we have in view the bacterial flora may be roughly divided into two classes—those which mainly attack and decompose carbohydrates, and those that mainly attack proteins or their hydrolysed products. The action of the former class is called fermentation, of the latter putrefaction.

During the process of excessive *fermentation* abnormal quantities of alcohol and of lactic, acetic, butyric, formic or oxalic acid may be formed, one of the products usually predominating. The acidity of the urine may be increased, as the acids if not oxidised combine with sodium and potassium, which deprives the phosphoric and sulphuric acid of some of their bases; but if excessive carbonic acid is at the same time formed in the gut, this tendency is antagonised, and the urine may even become alkaline. If the formation is due to an excessive or mainly carbohydrate dietary micturition is apt to become more frequent and the urine is increased in amount, unless diarrhœa accompanies it, when it will become more concentrated and more acid.

Gastric fermentation accompanied by a large carbohydrate dietary not infrequently leads to increased tissue waste, with a consequent increase in the uric acid output.

Alcohol and the organic acids themselves are, however, not found in any but minute traces in the urine, with the exception of oxalic acid, for they are readily oxidised in the body. Oxalates, if not due to excess of spinach, rhubarb, etc., denote in any but minute traces a pathological condition, and indicate gastro-intestinal fermentation. The condition has been produced experimentally by Baldwin,[1] who fed dogs with large quantities of cane sugar or glucose and meat, substances that in themselves contain no oxalic acid. Excessive gastritis with fermentation and pronounced oxaluria was the result. Oxalic acid was also produced in considerable amount by inoculating a medium of sugar and albumoses with material from the gastric contents. Gastric dilation, besides lending itself to oxaluria, may cause an acidosis, which, though mild, is very persistent.

The measure of acidosis can be made in a very few minutes in the following way:—20 c.c. of urine to which about a gram of potassium oxalate has been added are neutralised with $\frac{N}{10}$ NaOH, using phenolphthalein as an indicator. The mixture is then treated with 5 c.c. of formalin neutralised in the same way. The latter forms a compound with the ammonia, which is combined with the acids present, and thus the acids are liberated. Titration again with $\frac{N}{10}$ NaOH will indicate the amount of acidosis, and calculations will give the amount per cent. either in the terms of acid or of ammonia present.

The graver forms of acidosis do not, however, enter into our subject as they are metabolic disturbances rather than intestinal.

*Putrefaction*, or the excessive breaking-down of protein, is the result mainly of anaerobic bacteria. The intestinal canal, which may be regarded as a long oxygen-free or anaerobic tube, lends itself readily to this type of organism.

The hydrolysis produced by the digestive juices and the protein-splitting of bacteria follow very similar lines of cleavage, the similarity being due to the fact that the simple protein molecule is a large complex of amino-acids united together by their basic and acid groupings. The chemical action of the proteolytic enzymes of the intestine hydrolyses the complex protein food into albumoses and peptones, and peptone being soluble is rapidly absorbed. *In vitro* further splitting to amino-acids takes place, but this does not occur to a large extent in the living organism.

The action of the proteolytic bacteria, on the other hand, is a process of reduction. Oxygen is necessary for bacterial activity; and so, although the lines of cleavage are similar, the resulting products are different. Cleavage is extended further, and many substances useless for protoplasmic absorption are formed, many of them being actual poisons.

The amino-acids that enter into the composition of a protein molecule belong to three main groups. Firstly, we get the amino-acids of the fatty acid series. These contain one or two amino-acid groupings, the latter being known as the hexone bases. Secondly, there are the amino-acids of the aromatic series; and thirdly, there is cystin, a diamino-acid containing sulphur.

When proteolysis is excessive the fatty-acid series are to a large extent broken up to the simple constituents of $CO_2$, $H_2O$, $NH_3$, etc. From the $CO_2$ and $NH_3$ carbonate of ammonia is formed, which is synthesised into urea in the liver. The higher compounds are, however, resynthesised by the absorptive epithelium into bodies useful for protein formation; so that as far as the fatty acid series of amino-acids are concerned, we have no urinary index of the condition, except in cases of grave hepatic inadequacy. With the aromatics, however, the case is different, and in many cases they act as a valuable guide as to the amount of putrefaction occurring.

The chief aromatic constituents of the protein molecule are phenylalanin, tyrosin and tryptophane.

*Phenylalanin.*

*Tyrosin.*

*Tryptophane.*

These substances do not appear in the urine under ordinary circumstances, tyrosin does so only in cases of acute yellow atrophy and allied conditions, and once absorbed either as such or combined in protein, they become completely oxidised in the tissues, the benzene ring being split open. It is only in alkaptonurics that this does not occur, and under these circumstances the phenylalanin and tyrosin radicles appear in the urine as uroleucic and homogentisic acids.

When, however, intestinal putrefaction occurs, a more profound hydrolysis is associated with reduction, and bodies are formed in the gut and absorbed by the mucosa which are either protoplasmic poisons or substances which cannot be assimilated or made use of by the organism. These substances, if poisonous, are neutralised in the liver, and to a small extent in the tissues besides, to harmless aromatic sulphates or glycuronates, and are then excreted along with the non-toxic ones by the kidneys. As a general rule the greater portion of the aromatic products of putrefaction appear in the urine as sulphates.

Sulphates occur in the urine in two forms, the preformed or neutral sulphates and the ethereal. The former are principally formed from the products of tissue metabolism, and are practically proportioned to the nitrogenous output; the latter are usually almost entirely the products of intestinal putrefaction. Hence, if a quantitative estimate is made of both the preformed and the aromatic sulphates, and the ratio of the two is compared, an approximate estimate can be made of metabolism in general and of the extent of putrefaction, and the ratio which they bear to one another.

In health the daily output of the ethereal sulphates is from

.1 to .3 gram, reckoned as sulphuric acid, and the neutral sulphates from 1 to 3 grams; the ratio of the two is $\frac{1}{10}$ to $\frac{1}{16}$. In disease involving putrefaction the ratio may rise to $\frac{1}{5}$ or $\frac{1}{7}$ and even to $\frac{1}{1}$, or to a quotient greater than 1.

*Quantitative Estimation of Sulphates in Urine.*

*Total sulphates,* preformed and ethereal—

10 c.c. of urine (accurate)
10 c.c. of pure HCl.
20 c.c. of distilled water are put into a flask and boiled gently for half an hour. A funnel is placed in the neck of the flask to act as a condenser. The mixture is then cooled and 10 c.c. of 5 per cent. $BaCl_2$ is added. The barium sulphate is then treated and weighed as below.

*Inorganic sulphates*—

10 c.c. of urine (accurate)
10 c.c. of pure HCl.
20 c.c. of distilled water
10 c.c. of 5 per cent. $BaCl_2$

are put into a flask and the mixture is then allowed to stand for half an hour.

A small Gooch crucible is meanwhile taken, and a watery mixture of refined asbestos is placed inside. The crucible is placed on the water pump to express the water, so that an even film of asbestos lies on the surface of the perforations. The crucible is then carefully dried, gradually heated, then placed in a blow-pipe flame, and then into a dessicator. After allowing to cool it is carefully weighed. The total sulphates are now filtered through it on a water pump, care being taken always to have the crucible at least half full of fluid until the end. Any remaining traces of sediment are washed out of the flask and added.

The crucible is then dried, warmed, and placed under the blow flame as before, care being taken not to allow the flame to play on the inner surface of the crucible. After cooling it is weighed as before, and the difference in weight gives the total sulphates.

The inorganic sulphates are now treated in the same manner, the same crucible being used.

By subtracting the inorganic sulphates from the total the weight of the organic sulphates can be determined.

*Example.*

| | | |
|---|---|---|
| Weight of crucible .. .. | 13.927 grams | Difference=0.075 |
| Weight on addition of total sulphates .. .. .. | 14.002 ,, | |
| Weight on addition of inorganic sulphates .. | 14.069 ,, | Difference=0.067 |

Total sulphates=0.075 gram. in 10 c.c. of urine.
=0.75 per cent.
Inorganic ,, =0.067 gram. in 10 c.c. of urine.
=0.67 per cent.
Organic ,, =0.008
=0.08 per cent.

$$\text{Index} = \frac{\text{Organic}}{\text{Inorganic}} = \frac{0.08}{0.67} = \tfrac{1}{8}$$

If total urine for 24 hours=1,000 c.c.

Organic sulphates=0.08 per cent.=0.08 × 10=0.8 gram.

Reckoned as barium sulphate=0.33 gram. $H_2SO_4$.

Inorganic sulphates=0.67 per cent.=0.67 × 10=6.7 grams.

Reckoned as barium sulphate=2.76 grams. $H_2SO_4$.

The chief aromatics found in the urine are indican or indoxyl sulphate of potash, skatoxyl sulphate of potash, indol acetic acid—all derived from tryptophane—and the phenol and cresol sulphates—derived in the most part probably from tyrosin. In some cases glycuronates are found, especially if the supply of sulphuric acid runs short. The phenol and cresol derivatives are usually found in the urine only in minute quantities. They are usually increased when there is a general increase in the ethereal sulphates, such as occurs in certain cases of intestinal obstruction and peritonitis, also in anæmias, diabetes, typhoid and general cachectic conditions. Occasionally the quantity is increased in proportionally much larger amount, but no special significance has so far been attached to this. If present in large quantity they will react to Millon's reagent applied directly to the urine.

The tryptophane derivatives have been more carefully studied, as they lend themselves peculiarly to colour reactions, and in many cases some shrewd idea of the particular nature and the type of organism that is causing the putrefaction can be obtained .

The two primary katabolic products of tryptophane on undergoing bacterial cleavage are indol-propionic acid and indol-acetic acid. The former is unstable, and from it are derived indol and skatol, which are in part absorbed, held up, chiefly by the liver, and after hydration to indoxyl and skatoxyl are combined with sulphuric acid, and are then excreted in the urine as the potassium salts, indican and skatoxyl sulphate of soda. Indol-acetic acid is, on the other hand, much more stable, and as we shall presently see is excreted by the kidneys unchanged.

*Tryptophane.*

*Indol Propionic Acid.*

*Indol Acetic Acid.*

*Skatol.*

*Indol.*

*Skatoxyl.*

*Indoxyl.*

*Potassium Skatoxyl Sulphate.*

*Potassium Indoxyl Sulphate (Indican).*

The best-known tests for indican consist in treating it with some oxidising agent in acid solution and producing a deposit of indigo blue. The simplest way of performing the test is as follows :—Put about 5 c.c. of urine in a test tube and about 3 c.c. of HCl in another. Slant the acid HCl test tube, and place in the mouth of it a grain or two of bleaching powder. Now run the HCl gently over the bleaching powder into the test tube with urine. In a short time a well-marked blue ring will be formed if indican is present. As indigo is most insoluble in water and fairly soluble in chloroform it will be all taken up by chloroform if 2 or 3 c.c. are now added. The intensity of the blue is a rough indication of the quantity of indican present. Strauss' test is very similar, and consists in first acidifying the urine with lead acetate, filtering and adding equal quantities of HCl with a trace of ferric chloride in it and then chloroform.

This is the method that I am using for the quantitative estimation of indican :—

20 c.c. of urine are acidified with 5 c.c. of weak solution of lead acetate. 20 per cent. is advised, but I find this sometimes too strong. 10 c.c. of this, equivalent to 8 c.c. of urine, are treated with 10 c.c. of Obermeyer's solution of HCl. and ferric chloride, and with about 5 c.c. of chloroform are put into a separator. The mixture is well shaken two or three times and the chloroform allowed to settle, and then run off. More chloroform is added and separated, and the process is repeated two or three times until the chloroform remains uncoloured. After noting the quantity of the chloroform extract, a measured quantity is placed in the Veley's colorimeter and measured against a chloroform extract of known strength. From this a twenty-four hours' estimation of indican can be made.

Herter's classification [2] of the main types of intestinal putrefaction has now been generally accepted. These are the indolic, the saccharo-butyric, and the combined indolic and saccharo-butyric. The classification is a generalisation only, and should be used as a starting-ground for investigation rather than as a finality in classification, a matter he would have been the first to emphasise. However, his *indolic* type is based on the fact that a large quantity of indol is formed in the intestine and a more or less proportional quantity of indican is found in the urine.

Indol is produced in quantity either through the ascent of the colon bacilli into the small intestine, where they have free access to the peptones, or through the descent of native proteids to the large intestine, where they are attacked by the combined action of the putrefactive anaerobes and the colon bacilli, and any factors that produce either of these conditions are such as lead to pronounced indicanuria.

From what I have said it can readily be gathered that excess of or indigestible forms of protein are more likely to lead to indicanuria than moderation and the taking of proteins that are rapidly absorbed.

Indicanuria in youth can generally be easily treated by dietary and mode of life ; moreover, its effects are not injurious, as the margin of compensation by oxidation in the tissues is large. With age this is not so easy, nor is the compensatory mechanism so perfect. Prolonged indicanuria, especially if associated with the saccharo-butyric type of putrefaction, frequently leads to intractable neurasthenia. Lee[3] has proved experimentally that both indol and skatol have a fatiguing effect on muscle, a fact which may have some significance in this respect ; but it is, on the other hand, quite possible for debility to be the cause of indicanuria. If the alimentary secretions are for any reason feeble, proteolysis, as will be evident from what I have already said, will become more marked, for there will be more pabulum for the bacteria to deal with. Constipation may or may not lead to increased production. If the lodgment is well down in the sigmoid indican need not necessarily be increased unless it leads to stasis higher up.

The measure of indican is not always proportional to the amount of putrefaction or to the quantity of ethereal sulphates. As I have indicated, the phenol derivatives may at times be excessive. Indol-acetic acid may also to a large extent take its place, for it is rare to find both indican and indol-acetic acid marked in the same person, so that in cases of marked indol-aceturia the reliability of the indican test is impaired.

The test[4] for indol-acetic acid in the urine depends on two things—the presence of a nitrite in small or minute quantities and the presence of HCl. If the urine has stood for twelve hours or so nitrites are usually present—the product of nitrifying bacteria.

Strong HCl. is added to an equal quantity of urine, and a rose-red colour should appear. If it does not, the addition of a drop or two of .2 per cent. potassium nitrite will bring it out. Should the presence of indigo obscure the reaction, both the indigo blue and the indigo red can be readily extracted with chloroform. The supernatant fluid can than be pipetted off and treated with amyl alcohol, which will take up the indol-acetic compound urorosein, as it is called. The precise nature of the reaction is not known.

There are, however, other ethereal salts that on the addition of HCl. form a red body, notably skatol red. They can both be extracted with amyl alcohol, and both have an identical spectroscopic band between D and E. They differ, in that urorosein has a tint which is rose colour and is lighter and brighter than skatol red, which is redder. Urorosein is readily soluble in water, skatol red little so. The former is insoluble in ether and chloroform, the latter somewhat soluble. Administration of skatol to a dog will produce the skatol red reaction in urine and not the urorosein. The colouring matters derived from bile pigment may also give a somewhat similar HCl. reaction, but they can be thrown out by precipitation with lead acetate in dilute solution.

The significance of indolaceturia is not yet known, nor why it should supplant the indican and *vice versa*. In testing for ethereal bodies it should never be neglected. The clinical states that it is chiefly associated with are diabetes, typhoid fever, and chronic enteritis. It is marked in certain cases of jaundice and pulmonary tuberculosis. It has been noted in the vomiting of pregnancy, and it is generally present if there is any intestinal putrefaction.

One of the most marked cases described was of a boy of seven, whose skeletal and muscular development was severely retarded, and whose abdomen was markedly protuberant from long-standing distension with gases.

The paradimethylamidobenzaldehyde[5] reactions with the tryptophane derivatives are full of interest, but our knowledge of them is far from complete. Added to urine with HCl, with or without the aid of heat, a cherry-red colour is formed, which on the addition of a weak alkali to neutralise turns into a beautiful carmine. If amyl alcohol is now added, it takes up nearly the whole of the colouring matter. This reaction is influenced in several different ways, so that its full significance is not at present completely understood. It depends partly on the presence of urobilinogen. It is augmented by the feeding with or injection of skatol, or by the presence of much skatol in the fæces and *vice versa*. It is uninfluenced by administration of indol. Feeding with red meat increases it, but previous extraction of all the colouring matter diminishes.

Herter observed that the reaction corresponds in its intensity with the urorosein reaction after skatol administration, and he was disposed to think that the absorption of skatol from the intestine is a common cause of the aldehyde reaction.

An exact knowledge of the chemical processes produced in our alimentary tract, of the influence which the products absorbed have on our system, how they aid in causing disease and senility, are subjects of the utmost importance to our profession. It may be many years before we can gauge their true significance, but I feel convinced that a more regular examination of the urine for the bacterial by-products of the protein molecule will go some way in hastening the unravelling of these knotty problems.

REFERENCES.

1 *J. Exper. Med.*, 1900, v. 617.
2 "Common Bacterial Infections of the Digestive Tract," 1907, p. 278.
3 *Ibid.*, p. 255.
4 *J. Biol. Chem.*, 1908, iv. 101, 239.
*J. Am. M. Ass.*, 1908, l. 1959.
5 *J. Biol. Chem.*, 1906, i. 415.

*General.*

Herter, "Common Bacterial Infections of the Digestive Tract," 1907.
Herter, "Lectures on Chemical Pathology," 1902.
Wells, "Chemical Pathology," 1907.

## Figures and Tables

**Figure f1:**
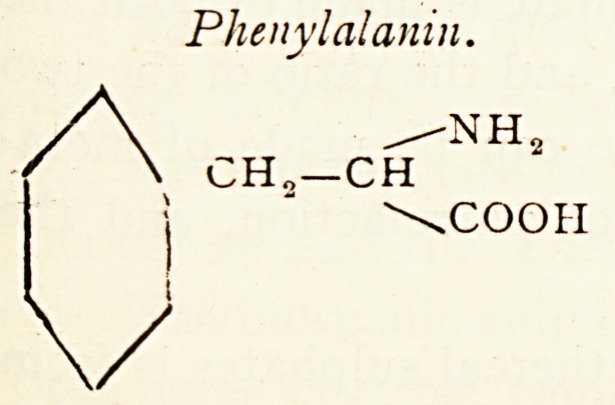


**Figure f2:**
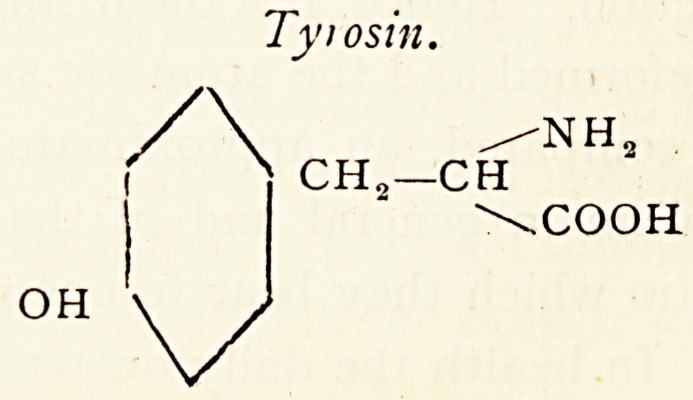


**Figure f3:**
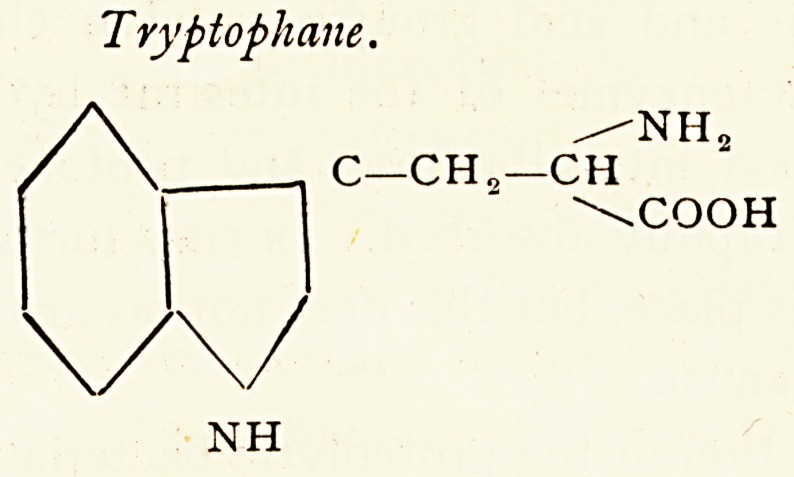


**Figure f4:**
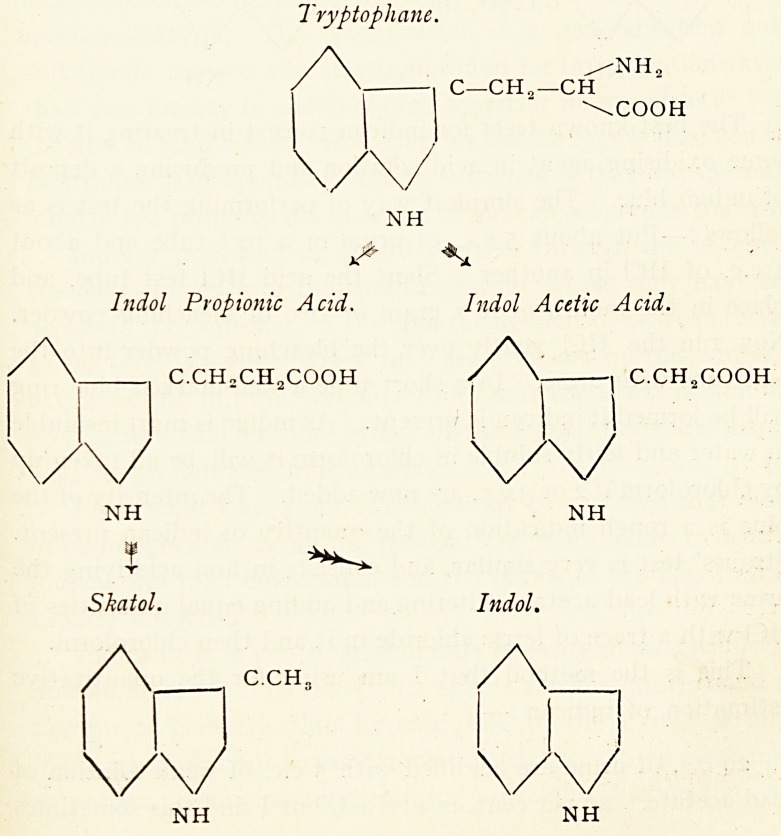


**Figure f5:**